# Whole-Genome Sequencing Reveals a Novel *GATA2* Mutation in Lower-Grade Glioma: Bioinformatics Analysis of Functional and Therapeutic Implications

**DOI:** 10.3390/cancers17203338

**Published:** 2025-10-16

**Authors:** Handoko, Vincent Lau, Eka Susanto, Renindra Ananda Aman, Didik Setyo Heriyanto, Soehartati A. Gondhowiardjo

**Affiliations:** 1 Department of Radiation Oncology, Cipto Mangunkusumo National General Hospital, Jakarta 10430, Indonesia; handoko12@ui.ac.id (H.); vincent.lau@ui.ac.id (V.L.); 2 Faculty of Medicine, Universitas Indonesia, Jakarta 10430, Indonesia; 3 Department of Anatomical Pathology, Faculty of Medicine, Universitas Indonesia, Dr. Cipto Mangunkusumo Hospital, Jakarta 10430, Indonesia; eka.susanto01@ui.ac.id; 4 Department of Neurosurgery, Faculty of Medicine, Universitas Indonesia, Dr. Cipto Mangunkusumo General National Hospital, Jakarta 10430, Indonesia; reninananda.aman@ui.ac.id; 5 Department of Anatomical Pathology, Faculty of Medicine, Public Health and Nursing, Universitas Gadjah Mada, Dr. Sardjito General Hospital, Yogyakarta 55284, Indonesia; didik_setyoheriyanto@mail.ugm.ac.id; 6 Collaboration Research Center for Precision Oncology Based Omics—PKR PrOmics, Yogyakarta 55284, Indonesia

**Keywords:** lower-grade glioma, *GATA2*, WGS, computational analysis, precision medicine

## Abstract

Background/Objectives: Lower-grade gliomas, particularly *IDH*-mutant astrocytomas, represent a distinct molecular subtype with unique therapeutic challenges. Whole-genome sequencing (WGS) plays a crucial role in uncovering genetic alterations that drive glioma pathogenesis and therapeutic resistance. This study identifies and evaluates a novel *GATA2* p.Arg396Trp mutation in a clinical sample of lower-grade glioma, assessing its structural impact and implications for drug binding. Methods: A WHO Grade II astrocytoma specimen from a 33-year-old female patient was analyzed using WGS with Oxford Nanopore sequencing, followed by comprehensive bioinformatics processing to identify genomic variants. The *GATA2* p.Arg396Trp mutation was evaluated using protein modeling, structural analysis, and pathogenicity prediction tools. Drug affinity analysis was conducted using molecular docking simulations to assess the computational impact of the mutation on drug binding. Results: The *GATA2* p.Arg396Trp mutation was identified as a computationally predicted pathogenic variant, potentially disrupting protein interactions within critical functional domains. Structural analysis revealed altered binding dynamics with key anti-neoplastic agents, suggesting potential implications for therapeutic response. These findings represent computational predictions requiring experimental validation. Conclusions: Our preliminary findings suggest a potential role of the *GATA2* p.Arg396Trp mutation in lower-grade glioma pathogenesis. The mutation predicted impact on transcriptional regulation and drug affinity suggests *GATA2* as a possible biomarker candidate. Extensive experimental validation in larger patient cohorts is needed to establish clinical relevance and explore targeted therapeutic strategies.

## Simple Summary

Lower-grade gliomas represent a distinct molecular subtype of brain tumors with unique therapeutic challenges. This study investigates a novel genetic mutation, p.Arg396Trp, in the *GATA2* gene, identified in an *IDH*-mutant astrocytoma patient through whole-genome sequencing. Using comprehensive computational methods, we examined how this mutation affects protein structure and interaction with cancer drugs. Our findings suggest that the mutation is pathogenic and may alter drug binding, potentially influencing treatment effectiveness. This research highlights the importance of genetic screening in lower-grade gliomas and suggests that *GATA2* could be a potential biomarker for precision medicine approaches.

## 1. Introduction

Gliomas represent a heterogeneous group of primary brain tumors with distinct molecular characteristics and clinical behaviors. The 2021 World Health Organization (WHO) Classification of Tumors of the Central Nervous System (CNS5) has fundamentally transformed glioma diagnosis by integrating molecular markers with histological features, emphasizing the critical importance of genetic profiling in clinical decision-making [1]. Lower-grade gliomas (WHO grades II and III) constitute approximately 15–20% of all primary brain tumors and are characterized by slower growth patterns compared to glioblastoma, yet they inevitably progress to higher-grade malignancies in most cases [2,3].

The molecular classification of gliomas has revealed fundamental distinctions between IDH-mutant and IDH-wildtype tumors, with IDH-mutant lower-grade gliomas representing a distinct entity with unique therapeutic vulnerabilities and prognostic implications [4]. Recent comprehensive genomic studies have identified key molecular alterations in lower-grade gliomas, including mutations in IDH1/2, TP53, ATRX, and CIC genes, along with 1p/19q codeletion in oligodendrogliomas [5,6]. These molecular insights have enabled more precise prognostic stratification and are increasingly guiding therapeutic decisions in clinical practice.

Whole-genome sequencing (WGS) has emerged as a powerful tool for comprehensive genomic characterization of gliomas, offering unprecedented insights into the mutational landscape and enabling the identification of rare but potentially significant genetic alterations [7,8]. Recent studies utilizing WGS in glioma cohorts have revealed novel driver mutations, structural variants, and non-coding regulatory alterations that contribute to tumor pathogenesis and therapeutic resistance [9,10]. The integration of WGS data with clinical outcomes has facilitated the development of precision medicine approaches, allowing for personalized treatment strategies based on individual tumor genomic profiles.

Transcription factors play crucial roles in glioma pathogenesis by regulating gene expression programs that control cell proliferation, differentiation, and survival [11]. *GATA2*, a zinc finger transcription factor traditionally associated with hematopoietic development, has recently emerged as an important regulator in various solid tumors, including brain tumors [12,13]. Fu et al. demonstrated that *GATA2* regulates constitutive *PD-L1* and *PD-L2* expression in brain tumors, suggesting its involvement in immune evasion mechanisms [14]. Furthermore, Wang et al. showed that *GATA2* promotes glioma progression through *EGFR/ERK/Elk-1* pathway activation, establishing its oncogenic role in glioma biology [15].

To our knowledge, this is the first report of the *GATA2* p.Arg396Trp mutation in a patient with lower-grade glioma. Our study provides a comprehensive computational framework for assessing the functional and therapeutic implications of rare mutations in transcription factors, which may serve as a model for precision medicine approaches in neuro-oncology. Through integrated genomic analysis, structural modeling, and drug affinity prediction, we aim to elucidate the potential pathogenic mechanisms of this novel *GATA2* variant and its implications for therapeutic targeting in lower-grade gliomas.

## 2. Materials and Methods

A fresh frozen glioma specimen was obtained from a 33-year-old female patient who presented with new-onset seizures and was found to have a left frontal lobe mass on neuroimaging. The sample was procured from the biobank at the Department of Radiation Oncology, Cipto Mangunkusumo National General Hospital, with ethical approval from the Faculty of Medicine, Universitas Indonesia (No. KET-289/UN2.F1/ETIK/PPM.00.02/2024), and the patient provided informed consent for genetic testing.

During a subtotal tumor resection, a research sample of approximately 45 mg was collected and cryopreserved in a liquid nitrogen tank at −80 °C for subsequent molecular analysis. Histopathological examination by two independent pathologists confirmed a diagnosis of WHO Grade II astrocytoma.

Molecular analysis classified the tumor as an IDH-mutant astrocytoma under WHO CNS5 criteria, revealing a pathogenic IDH1 p.Arg132His (R132H) mutation, two ATRX variants of uncertain significance (p.Thr1755Ala and p.Glu891Gln), and a hypermethylated TERT promoter. Following surgery, the patient received adjuvant radiotherapy totaling 54 Gy in 30 fractions. At her 12-month follow-up, the disease remained stable.

### 2.1. DNA Extraction

Following histological confirmation, a 45 mg tissue specimen was processed for DNA extraction. The specimen was thawed and manually dissected in 100 μL of phosphate-buffered saline (PBS) using a sterile surgical knife. Mechanical shearing was performed with 250 μL PBS and ultrasonic sonication for 10–15 s, repeated until no visible aggregates remained. DNA extraction was performed using the QIAamp DNA Mini kit (QIAGEN, Hilden, Germany) following the manufacturer’s protocols. Quality assessment was performed using a NanoDrop Spectrophotometer (A260/A280 ratio > 1.8; Thermo Fisher Scientific, Waltham, MA, USA) and quantification with a Qubit Fluorometer (Thermo Fisher Scientific, Waltham, MA, USA). DNA integrity was verified by agarose gel electrophoresis.

### 2.2. DNA Library Preparation and DNA Sequencing

Library preparation was performed using the Oxford Nanopore Ligation Sequencing DNA V14 (SQK-LSK114) protocol without PCR amplification to minimize bias. Sequencing was conducted on Oxford Nanopore PromethION 2 Sequencing Unit Solo with PromethION R10.4.1 flow cell. The final library concentration was 50 fmol, as per the manufacturer’s specifications. Sequencing ran for 71 h until pore depletion, with library reloading performed twice when occupancy dropped to approximately 20%. Target coverage was >30× for reliable variant calling.

### 2.3. Bioinformatics: NanoPlot Analysis

The glioma samples yielded raw sequencing data in POD5 format. Basecalling was executed utilizing the EPI2ME ‘wf-basecalling’ pipeline (https://github.com/epi2me-labs/wf-basecalling; accessed on 12 June 2025), leading to the production of FASTQ files. The quality of the FASTQ data was evaluated using NanoPlot (https://github.com/wdecoster/NanoPlot; accessed on 12 June 2025) to verify the precision and dependability of the sequencing reads.

Raw sequencing data in POD5 format underwent basecalling using the EPI2ME wf-basecalling pipeline (v1.1.0) with a high-accuracy model. Quality assessment was performed using NanoPlot (v1.40.0) with a minimum quality score threshold of Q10. Sequence alignment to the GRCh38 reference genome was performed using the EPI2ME wf-alignment pipeline (v1.0.0) with minimap2 aligner. Alignment quality was assessed using samtools stats, requiring a minimum mapping quality of 20.

The sequencing data was subsequently aligned using the EPI2ME ‘wf-alignment’ pipeline (https://github.com/epi2me-labs/wf-alignment; accessed 12 June 2025) against the human reference genome GRCh38, obtained from the UCSC Genome Browser. The alignment results, stored in BAM format, were visualized using the Integrative Genomics Viewer (IGV) Desktop Application version 2.17.0 to assess mapping coverage and pinpoint mutation sites.

Copy number variation (CNV) analysis was conducted on the BAM files utilizing the EPI2ME ‘wf-human-variation’ CNV calling pipeline (https://github.com/epi2me-labs/wf-human-variation; accessed 12 June 2025). The CNV data were examined to identify regions of gene amplification or deletion linked to glioma. The functional consequences of gene gains and losses were assessed concerning glioma phenotypes.

Single-nucleotide variant (SNV) calling was performed using the EPI2ME wf-human-variation pipeline (v1.2.0) with the following parameters: minimum coverage depth 10X, variant allele frequency threshold 0.2, and quality score threshold 20. Variants were annotated using SnpEff (v5.0) against the Ensembl GRCh38.99 database. Pathogenicity prediction was performed using multiple algorithms, including SIFT, PolyPhen-2, MutationTaster, and CADD scores. Variants were classified according to ACMG guidelines, with pathogenic variants defined as those meeting criteria for likely pathogenic or pathogenic classification.

Identified SNVs annotated in the ClinVar database were categorized according to their significance in cancer-related pathways, specifically those associated with glioma, utilizing the KEGG database. The ShinyGO 0.80 tool (http://bioinformatics.sdstate.edu/go, accessed 22 January 2025) was employed to do target annotation of the ClinVar SNV results [16]. The annotation predominantly highlighted biological processes and Kyoto Encyclopedia of Genes and Genomes (KEGG) pathways. A graphical depiction of SNV types and their functional roles in pathways associated with glioma was created.

Potential driver mutations were meticulously curated by analyzing the biological functions of genes containing SNVs and their roles in glioma pathogenesis. A comprehensive visualization of the aligned sequencing data and mutations was conducted using IGV to verify structural variations and evaluate their co-localization with glioma-associated genes. The identified CNVs and SNVs were cross-referenced with the ENSEMBL database to ensure thorough chromosomal localization, gene functionality, and pathway associations.

### 2.4. Mutagenesis of the Modeled Protein

To assess the structural and functional effects of the p.Arg396Trp mutation in *GATA2*, the mutation was manually introduced into the AlphaFold-modeled *GATA2* protein using PyMOL software Version 3.0.4. The procedure began with the importation of the AlphaFold-generated *GATA2* structure into PyMOL for the visualization of the protein’s three-dimensional conformation. The specific residue to be mutated, arginine at position 396, was identified through careful inspection of the sequence viewer and direct selection of the corresponding residue within the structural model. Upon identifying the target residue, the mutagenesis tool in PyMOL was employed to enable the modification. The residue was substituted with tryptophan (Trp), indicating the p.Arg396Trp mutation. The manual mutation process facilitated accurate modification of the protein’s amino acid sequence, allowing for subsequent structural and functional analyses to investigate the effects of this substitution on drug binding and overall protein activity.

### 2.5. Mutation Taster Analysis

To evaluate the potential pathogenicity of the *GATA2* p.Arg396Trp (c.1186C>T) mutation, we employed MutationTaster, an established web-based tool that integrates multiple predictive algorithms to assess the functional consequences of genetic variants [17]. The analysis involved submitting the nucleotide substitution to the MutationTaster database, where it was compared against reference gene structures to determine its impact on protein function.

The mutation’s potential effect was assessed through several parameters, including evolutionary conservation, splice site alterations, and protein domain disruption. Conservation analysis examined whether the affected residue was highly conserved across species, indicating its functional importance. Additionally, the mutation’s location within key protein domains was analyzed using InterPro annotations, highlighting whether the substitution could disrupt critical functional regions. MutationTaster also assessed the likelihood of the mutation creating or altering splice donor or acceptor sites, which could impact mRNA processing and protein translation.

The final classification was based on a probability score ranging from 0 to 1, with values close to 1 suggesting a high likelihood of pathogenicity. The findings from MutationTaster were further cross-referenced with structural and bioinformatics analyses to contextualize the mutation’s potential role in glioma pathogenesis, particularly its implications for transcriptional regulation and drug binding.

### 2.6. HOPE Analysis

Have Our Protein Explained (HOPE) is a website-based tool that evaluates the structural and functional consequences of point mutations on protein architecture. We can either upload the mutated PDB file or employ sequence-based analysis to forecast the impact of mutations on the protein’s structure and function. The three-dimensional conformation of the protein in issue is determined. Data from this three-dimensional structure will be obtained via WHAT IF Web services, UniProt database, and the Reprof software. The structural data were obtained by the examination of the PDB. Information pertaining to this protein was obtained from the UniProt entry [18].

#### 2.6.1. Amino Acid Analysis

The analysis will evaluate the effects of the mutation on the following aspects: interactions mediated by the mutated residue; structural domains including the residue; modifications to this residue; and recorded variants linked to this residue. A concise conclusion based solely on the properties of amino acids is consistently expressed. If a 3D structure/model is available, the report will further include photographs and animations.

#### 2.6.2. Genomic Variants Analysis

We utilized dbNSFP to associate this mutation with a genomic variant. The score will range from 0.0 to 1.0; higher values indicate an increased likelihood of pathogenicity from the mutation.

MetaRNN and MetaRNN-indel are computational tools developed to assess the pathogenicity of nonsynonymous single-nucleotide variants (nsSNVs) and non-frameshift insertions and deletions (indels) in humans. These models leverage a comprehensive dataset that includes 28 high-level annotation metrics, combining 16 functional prediction tools and 8 conservation-based scores. Additionally, they incorporate allele frequency data from several major genomic resources. By utilizing a deep recurrent neural network (RNN), MetaRNN generates a probability score that predicts the likelihood of a variant being pathogenic. This data-driven approach improves the accuracy of mutation effect predictions, facilitating more reliable variant interpretation in genomic research [19].

dbNSFP is an extensive resource designed to assist in the functional prediction and annotation of nonsynonymous single-nucleotide variants (nsSNVs) and splicing-site SNVs (ssSNVs) in the human genome. The latest version, based on Gencode release 29 and Ensembl version 94, includes over 84 million variants and integrates scores from various computational algorithms used to assess pathogenicity. These algorithms evaluate the potential impact of variants on gene function, including tools for predicting disease-related changes. Additionally, the database provides multiple conservation metrics, such as phylogenetic scores, as well as allele frequency data from major genomic projects, including large-scale population studies. It also includes valuable gene annotations, functional information, gene expression profiles, and interaction data, offering comprehensive insights for genomic research and variant analysis [20,21].

#### 2.6.3. Domain Identification

InterPro domain analysis was performed to identify functionally significant regions within the protein. The identified domains were cross-checked against the mutation site to determine whether the mutation occurred within or near a critical functional area. Gene Ontology (GO) annotations for the protein were retrieved using HOPE’s automated mapping system and further verified through online databases. The functional annotations were categorized into two groups: specific GO terms, which included attributes such as DNA-binding transcription factor activity and zinc ion binding, and broader GO classifications, which encompassed biological processes like transcriptional regulation and molecular functions [18].

### 2.7. Drug Affinity Analysis

To evaluate the potential effects of specific mutations in genes on drug binding and to identify therapeutic agents for glioma, we performed an in silico drug affinity analysis utilizing DrugRep and CB-Dock2. DrugRep, a sophisticated online platform for virtual drug screening and repurposing, was employed to identify compounds with significant binding affinity to the *GATA2* protein, while CB-Dock2 was utilized to validate and enhance these results through cavity-specific docking simulations [22,23].

The three-dimensional conformation of the complete protein, encompassing the mutation, was generated utilizing AlphaFold. This structure served as the receptor for docking studies. In DrugRep, receptor-based screening was conducted by identifying potential drug-binding cavities on the protein utilizing the CurPocket algorithm. The region surrounding the mutation was chosen for analysis due to its potential impact on drug binding, based on the identified binding pockets. DrugRep subsequently conducted a virtual screening of drug libraries utilizing AutoDock Vina to forecast the binding affinities of diverse compounds to the chosen pocket. Docking parameters included the following: grid box dimensions 20 × 20 × 20 Å centered on the mutation site, exhaustiveness parameter set to 8, and energy range of 3 kcal/mol. Binding affinity scores were calculated as the lowest energy conformation from 9 poses generated per compound. The platform ranked the compounds based on docking scores, presenting the top 100 compounds in the output along with their interactive 3D conformations [22].

The docking results for the leading 100 compounds were contrasted between the wild-type and mutated gene models. Compounds that ranked in the top 100 for the wild type but were absent from the top 100 post-mutation underwent manual validation and additional analysis through cavity-specific docking simulations utilizing CB-Dock2. CB-Dock2 assessed binding conformations according to binding energies, while its interactive visualization tools enabled comprehensive analysis of receptor-ligand interactions. This analysis concentrated on fundamental molecular interactions, such as hydrogen bonding, hydrophobic interactions, and electrostatic forces, which are vital for stable drug binding. This method yielded profound insights into the effects of mutations on binding affinity and compound specificity, thereby improving the reliability of the docking outcomes [24]. The residue and compound molecular docking were then visualized using CB-Dock2 and Discovery Studio Visualizer

## 3. Results

### 3.1. NanoPlot Analysis

Whole-genome sequencing of the lower-grade glioma specimen produced high-quality data. The sequencing achieved a mean coverage of 35×, with 98.2% of the genome covered at a depth of at least 10×. Analysis of the sequencing data identified 4247 high-confidence variants, which consisted of 3891 single-nucleotide variants (SNVs) and 356 small insertions/deletions (indels). Of these, 127 variants were located within coding regions, comprising 89 missense, 23 synonymous, and 15 loss-of-function variants. The quality of the sequencing reads was validated using NanoPlot. The dataset included 4.2 million reads, totaling 42.5 billion bases, of which 90% (39.99 billion bases) were successfully aligned to the human reference genome. The reads demonstrated high accuracy, with a median percent identity of 98.9% and a median quality score of 19.7. The N50 read length was 18,230 bases, indicating a strong representation of long reads and confirming the reliability of the dataset for downstream analysis.

#### 3.1.1. Copy Number Variations

**Figure 1:**
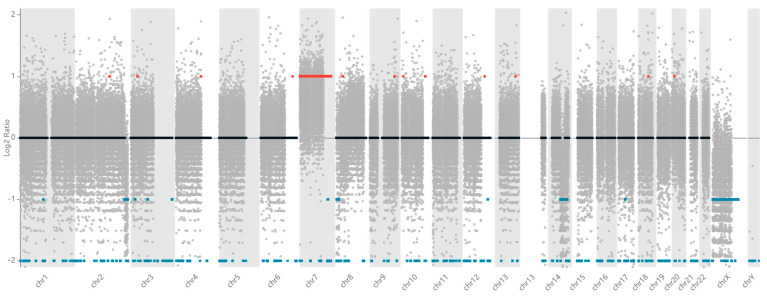
Genomic landscape of mutations identified by whole-genome sequencing. Red dots represent high-impact mutations (nonsense, frameshift, splice site variants), blue dots represent moderate-impact mutations (missense variants), gray dots represent low-impact variants (synonymous, intronic) and the black horizontal dashed line in the middle of the plot represents the baseline (log2 ratio = 0). The *GATA2* p.Arg396Trp mutation is highlighted with an arrow. Mutation frequency is plotted on the y-axis, with chromosomal position on the x-axis.

According to the CNV analysis, multiple chromosomal losses are observed across various chromosomes, as illustrated in Figure 1. Detailed information regarding each affected chromosome and gene, including their base positions, is provided in Table S1. This analysis was conducted on a clinical sample from an IDH-mutant glioma grade 2. Our findings align with existing literature and demonstrate consistency with the molecular subtype of glioma under investigation, which will be discussed in greater detail in the Discussion section.

#### 3.1.2. Single Nucleotide Variants

**Figure 2:**
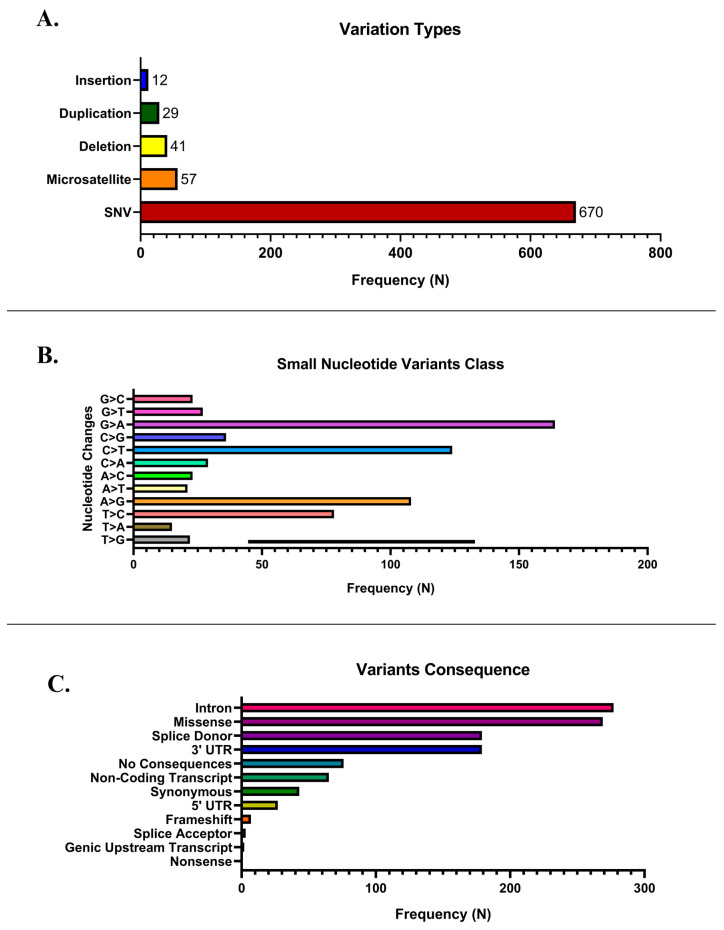
Summary of SNVs from an extensive whole-genome sequencing of a clinical glioma annotated to the ClinVar database. (**A**) Overview of the prevalence of SNVs and other variant types; (**B**) overview of the variants class of SNVs; (**C**) overview of the variants of consequences.

Genetic variations identified from the extensive whole-genome sequencing of a clinical glioma sample were annotated using the ClinVar database. SNVs are the most prevalent, with a frequency of 670, exceeding other variation types significantly. Single-nucleotide changes in the genetic landscape are a trend frequently observed in cancer and various genetic disorders. Microsatellites are the next most common (*n* = 57), followed by deletions (*n* = 41), duplications (*n* = 29), and insertions (*n* = 12). Figure 2A illustrates the distribution of these variants. These findings suggest that SNVs are likely the primary drivers of the observed genetic alterations in the dataset.

Figure 2B demonstrates the classification of SNVs based on nucleotide changes. The most frequently observed mutation is C>T transitions, with a frequency exceeding 180 occurrences. This is consistent with known patterns of SNVs, where C>T transitions are commonly associated with deamination of methylated cytosines, a hallmark of mutational processes such as aging or exposure to mutagens like ultraviolet radiation. Other significant mutations include T>C transitions and G>A transitions, highlighting the diversity of nucleotide changes contributing to the mutational landscape in the study population. Conversely, transversions such as C>A, A>T, and T>A were observed less frequently, reflecting their typically lower occurrence due to higher energetic costs or less favorable mutational mechanisms.

Figure 2C depicts the classification of genetic variants by their functional consequences. The most prevalent variant type occurs in intronic regions, accounting for the highest frequency (>250 occurrences). This observation is consistent with the earlier results showing frequent C>T transitions, as intronic regions are often susceptible to such mutations. Intronic variants, while non-coding, may affect splicing mechanisms and regulatory elements, potentially altering gene expression. Missense mutations are the second most common variant type, with a frequency similar to that of intronic variants. Missense mutations result in amino acid substitutions and are crucial for understanding pathogenic mechanisms, as they may alter protein structure and function. This finding underscores the potential for these mutations to contribute to disease etiology, particularly in cancer genomics. Variants affecting splice donor sites and the 3′ UTR follow in frequency, emphasizing the impact of mutations on post-transcriptional regulation and splicing fidelity. These mutations could disrupt mRNA processing, leading to aberrant protein production or stability. Conversely, frameshift, splice acceptor, and nonsense mutations were relatively rare. Table S1 provides data for all variants.

#### 3.1.3. ClinVar Annotation

Glioma clinical sample WGS identified the top 15 entries of pathogenic or likely pathogenic variants in genes previously associated with disease phenotypes, as annotated by ClinVar (Table 1). Among the genes identified, several are well-known for their roles in various pathologies, including *IDH1*, *TP53*, and *MTHFR*, which have been extensively implicated in glioma or other cancer pathways. For instance, mutations in *IDH1* (c.395G>A, p.Arg132His) and TP53 (c.559+1G>T) are hallmark alterations frequently reported in gliomas. Similarly, *HNF1A* and *MAPK7* variants contribute to oncogenic pathways and cellular dysregulation.

#### 3.1.4. KEGG Pathway Enrichment Analysis

**Figure 3:**
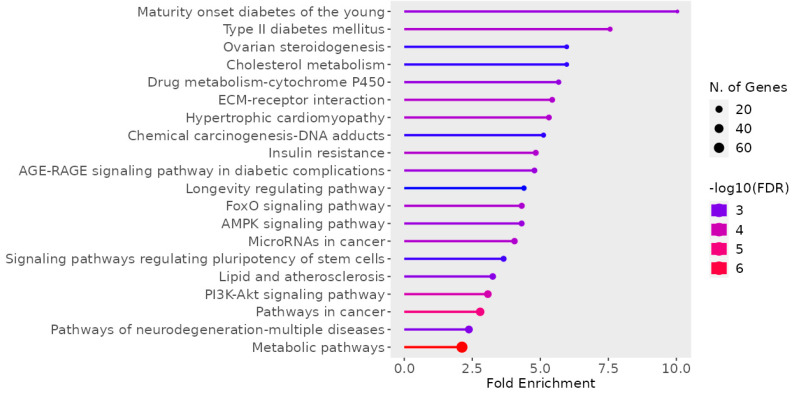
KEGG pathway enrichment analysis for SNVs annotated from ClinVar in clinical glioma patients.

**Figure 4:**
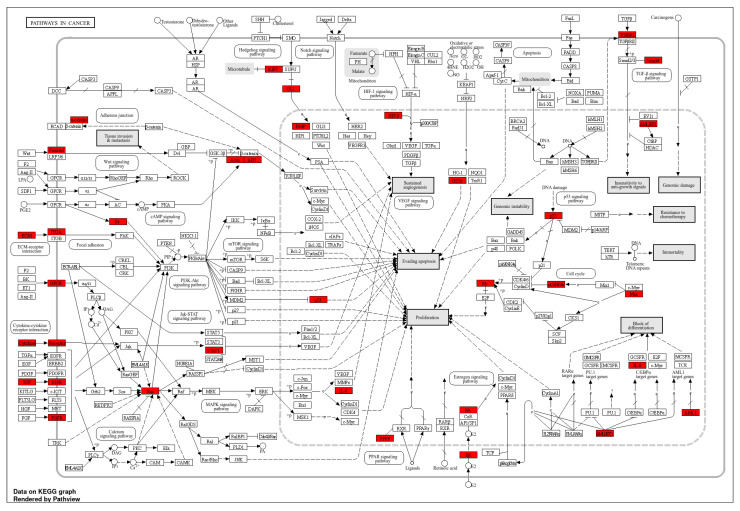
KEGG cancer-related pathways include the *PI3K-Akt* signaling system, *AMPK* signaling pathway, and pathways regulating pluripotency of stem cells. The red boxes denote genes from our dataset identified in ClinVar. Solid arrows indicate direct molecular interactions or activation/inhibition relationships, while dashed arrows represent indirect or regulatory interactions between signaling components and pathways.

The KEGG pathway enrichment analysis of SNVs annotated from ClinVar in clinical glioma patients identifies numerous biologically relevant pathways that may be critical in glioma genesis and progression. The most enriched pathways are metabolic and cancer-related pathways, which exhibit noticeable fold enrichment and are presented in Figure 3. These pathways, especially metabolic activities, underscore the significant influence of gliomas on cellular metabolism, potentially indicating tumor adaptability to fulfill its energy and biosynthetic requirements. Furthermore, cancer-related pathways include the *PI3K-Akt* signaling system, *AMPK* signaling pathway, and pathways regulating the pluripotency of stem cells exhibit some enrichment, highlighting their possible mechanistic connections to glioma biology (Figure 4).

Notably, the analysis revealed a mutation in the *GATA2* gene (c.1186C>T, p.Arg396Trp), classified as likely pathogenic. This finding is particularly significant because, unlike the other genes in the top 15, *GATA2* has not previously been reported in glioma clinical samples. The *GATA2* gene was not included among the genes documented in any KEGG-registered cancer-related pathway. *GATA2*, a transcription factor crucial for hematopoietic and vascular development, has been implicated in various hematological malignancies and immune-related disorders but remains underexplored in glioma. This discovery highlights the potential of *GATA2* as a novel candidate gene in glioma pathogenesis, warranting further investigation.

The *GATA2* p.Arg396Trp mutation was identified as a novel variant not previously reported in glioma literature. This mutation occurs in exon 6 (transcript NM_032638.5) or exon 7 (transcript NM_001145661.2). This discrepancy in exon numbering arises from different transcript reference sequences but does not affect the protein-level mutation designation (p.Arg396Trp). The mutation is located within the C-terminal zinc finger domain, a critical region for DNA binding and transcriptional regulation.

Multiple pathogenicity prediction algorithms classified the *GATA2* p.Arg396Trp mutation as likely pathogenic. MutationTaster assigned a probability score of 0.999, CADD score was 28.4 (>20 indicating pathogenicity), and SIFT predicted the variant as deleterious (score 0.02). The arginine at position 396 is highly conserved across vertebrate species, supporting its functional importance. While this specific p.Arg396Trp variant has not been previously reported in any disease context, other pathogenic *GATA2* mutations are well-documented in hematologic disorders, particularly *GATA2* deficiency syndrome, where they typically disrupt zinc finger domain function and DNA binding capacity.

The presence of well-documented pathogenic mutations alongside this novel finding underscores the heterogeneity of glioma at the molecular level and provides new insights into potential genetic contributors to the disease. Here, we reported a comprehensive in silico analysis for this particular *GATA2* mutation.

### 3.2. Mutagenesis of the Modeled Protein

Our study utilized whole-genome sequencing and subsequent bioinformatics analysis, which identified a mutation in the *GATA2* gene, specifically NM_001145661.2:c.1186C>T, leading to an amino acid substitution of p.Arg396Trp in exon 7. The mutation was also confirmed as NM_032638.5:c.1186C>T, resulting in the identical substitution p.Arg396Trp, annotated in exon 6. This variation denotes a single nucleotide alteration from cytosine to thymine at position 1186 of the coding sequence, resulting in the substitution of arginine with tryptophan at position 396 of the protein. This mutation, situated within a vital functional domain of *GATA2*, may affect its transcriptional activity and subsequent regulatory functions.

**Figure 5:**
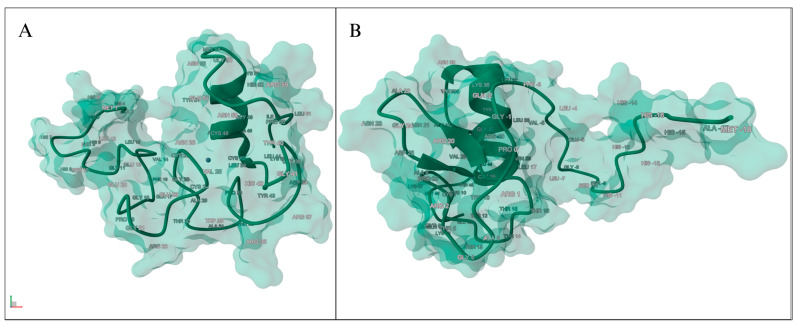
Human *GATA2* protein visualization with RCSB PDB. (**A**) Solution NMR structure of human *GATA2* C-terminal zinc finger domain, PDB: 5O9B. (**B**) Solution NMR structure of human *GATA2* N-terminal zinc finger domain, PDB: 6ZFV.

Our bioinformatics and in silico analysis employed existing structural data for the *GATA2* protein to evaluate the effects of the identified mutation p.Arg396Trp. We initially obtained Protein Data Bank (PDB) files 5O9B and 6ZFV [39,40], which correspond to the solution NMR structures of the C-terminal and N-terminal zinc finger domains of human *GATA2*, respectively (Figure 5). Although these structures offered significant insights into particular domains of the protein, their brief residue lengths constrained their effectiveness in illustrating the location and context of the p.Arg396Trp mutation, which is situated outside the modeled regions of these structures. Below, we can visualize the model using the RCSB Protein Data Bank (RCSB PDB) [41].

**Figure 6:**
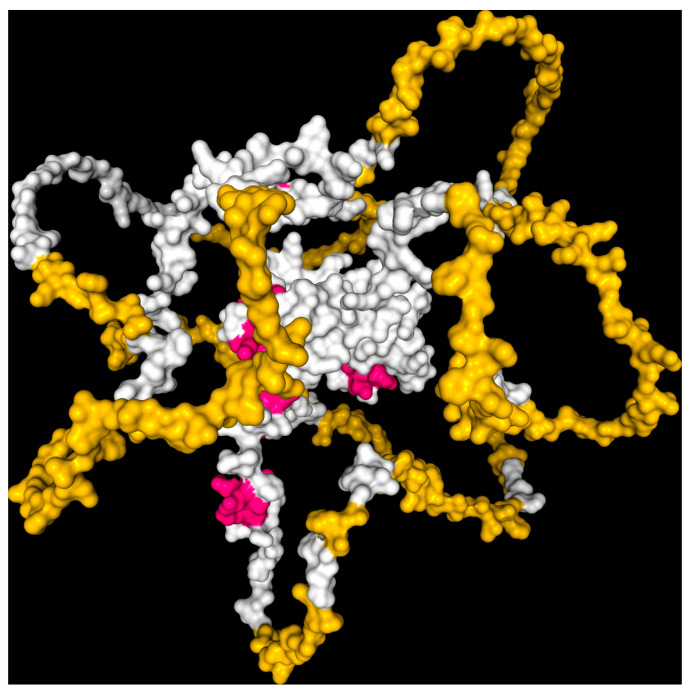
AlphaFold-2 modeled Human *GATA2* protein visualization with CB-Dock2. Molecular surface representation of the C-terminal and N-terminal zinc finger domains of human *GATA2.* The α-helices and β-sheets are shown in white, loop regions in yellow, and key residues involved in DNA binding or pathogenic mutations in magenta.

In order to address this limitation, we utilized the AlphaFold-2 Protein Structure Database to obtain a comprehensive model of the *GATA2* protein, allowing us to analyze the mutation within the context of the entire protein structure (Figure 6). This simulated model allowed us to assess the possible structural and functional ramifications of the p.Arg396Trp substitution, along with its effects on protein stability, DNA-binding capacity, and potential interactions with regulatory molecules or therapeutic agents. This thorough structural methodology was crucial for advancing subsequent in silico and drug affinity evaluations [42,43].

### 3.3. Mutation Taster Analysis

The mutation designated as NM_001145661:c.1186C>T (p.Arg396Trp) in the *GATA2* gene was evaluated with MutationTaster to ascertain its potential implications. MutationTaster categorized the mutation as disease-causing, supported by a high probability score of 0.99999992364159, showing strong confidence in its pathogenicity. This classification aligns with established evidence, as the variant is a documented disease mutation linked to pathogenic effects (dbSNP ID: rs800683).

The mutation entails a single nucleotide substitution, converting cytosine (C) to thymine (T) at position 1186 of the coding sequence, resulting in an amino acid substitution from arginine (Arg) to tryptophan (Trp) at position 396. This substitution occurs in a crucial region of the protein that does not modify its overall length but changes the amino acid sequence, potentially influencing protein function. The mutation has been previously recorded in databases like HGMD (CM116978) as a pathogenic variant, thereby reinforcing its association with disease. The mutation was not found in population databases such as ExAC or 1000G, indicating it is a rare variant rather than a prevalent polymorphism.

The *GATA2* mutation NM_032638:c.1186C>T (p.Arg396Trp) was also assessed using MutationTaster, which identified the variant as disease-causing with the exact probability score of 0.99999992364159, indicating strong confidence in its pathogenicity. The results from NM_032638 aligned with those of NM_001145661, as both transcript variants indicate the identical amino acid alteration (p.Arg396Trp) resulting from the c.1186C>T mutation, despite annotating distinct exons due to transcript variations (exon 6 in NM_032638 and exon 7 in NM_001145661).

MutationTaster verified that the mutation modifies the amino acid sequence without truncating the protein or inducing a frameshift, thereby maintaining the protein’s overall length while potentially impacting its functional domains. The results emphasize the infrequency and possible clinical importance of the p.Arg396Trp mutation in *GATA2*, indicating its probable involvement in disease pathogenesis and necessitating additional structural and functional investigations to clarify its biological effects. The findings suggest that the p.Arg396Trp mutation in *GATA2* likely has substantial implications for disease pathogenesis, necessitating further functional analysis to elucidate its role in gliomas.

### 3.4. HOPE Analysis

#### 3.4.1. Amino Acid Analysis

**Figure 7:**
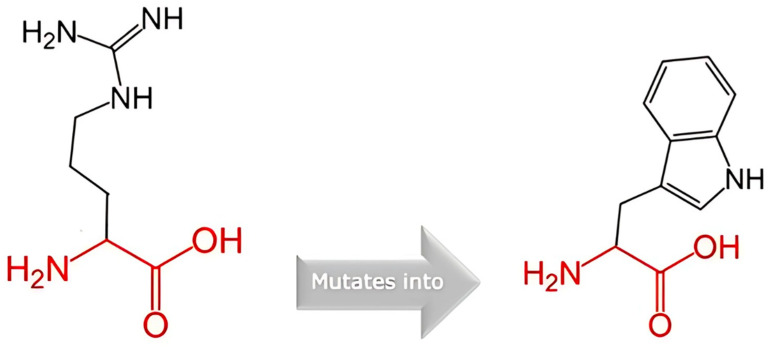
The schematic structures of the original amino acid (**left**) and the mutant amino acid (**right**).

Arginine was substituted with tryptophan at position 396. Figure 7 below depicts the schematic structures of the original amino acid (left) and the mutant amino acid (right). The backbone, common to all amino acids, is illustrated in red, whilst the side chain, distinctive to each amino acid, is represented in black. Each amino acid varies in dimensions, electrical charge, and hydrophobic characteristics. The mutant residue is bigger than the wild-type residue. Furthermore, the wild-type residue (arginine) possesses a positive charge, while the mutant residue (tryptophan) is neutral. Moreover, the mutant residue demonstrates enhanced hydrophobicity relative to the wild-type residue.

**Figure 8:**
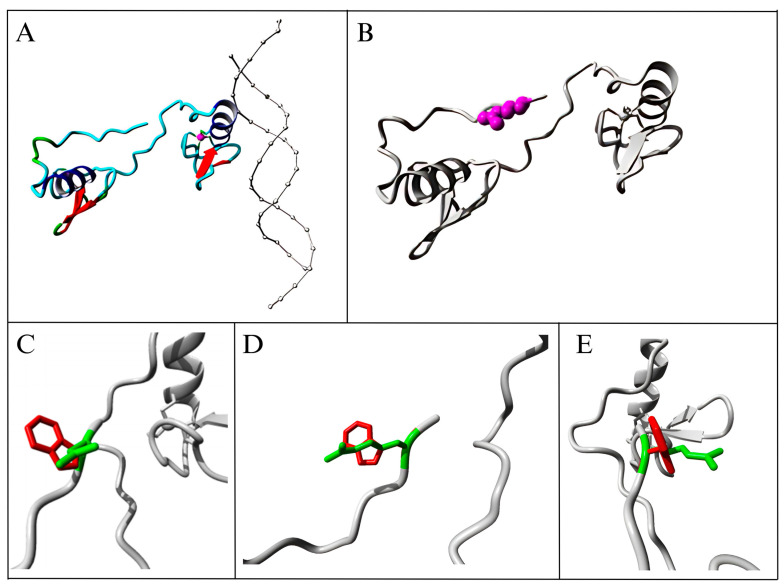
Overview of the protein in ribbon representation. (**A**) The protein is color-coded by element: α-helix is blue, β-strand is red, turn is green, 3/10 helix is yellow, and random coil is cyan. Other molecules in the complex are depicted in gray when present; (**B**) The protein is depicted in gray, while the side chain of the mutated residue is represented in magenta and illustrated as small spheres; (**C**–**E**) A close-up of the mutation, as observed from three distinct perspectives. The protein is shown in gray, with the side chains of the wild-type and mutant residues depicted in green and red, respectively.

The wild-type residue is extensively preserved; yet, other alternative residue types have been recognized at this location. No mutant residue or any other residue type with similar features was identified at this location in any homologous sequences. This mutation is probably detrimental to the protein according to conservation scores. Figure 8 illustrates the protein overview in ribbon representation.

#### 3.4.2. Genomic Variants Analysis

The MetaRNN score for this variant is 0.9318739. Hence, it has a high likelihood of pathogenicity.

#### 3.4.3. Domain Identification

The altered residue is located in a region vital for the protein’s activity and interacts with another domain that is equally crucial for its function. The mutation may interfere with the interaction between these domains, thereby compromising the protein’s function. The mutant residue is located in a domain essential for the protein’s function and interacts with residues in a distinct domain. This interaction may be essential for the protein’s correct function. The mutation may affect this connection and, thus, alter protein function.

The altered residue is located in a region essential for the protein’s function and interacts with another domain known for its binding role. The mutation may disrupt the connection between these domains, perhaps affecting signal transmission between them. The outcome of domain identification was displayed in Table 2.

### 3.5. Drug Affinity Analysis

An analysis of drug affinity was performed for the AlphaFold-modeled wild-type and p.Arg396Trp-mutated human *GATA2* proteins. Mutagenesis was performed with PyMOL as described in the method section. The detailed outcomes of the top 100 compound affinity analyses are presented in Table S1. The affinity differences among the compounds that consistently ranked in the top 100 across both models varied from −0.3 to +0.8. Significantly, four anti-neoplastic agents—Entrectinib, Rucaparib, Vemurafenib, and Brigatinib—were excluded from the top 100 in the mutated model, necessitating further examination of their modified binding characteristics. Currently, no clinically applicable *GATA2* inhibitors are available to serve as a control for this study. Given the clinical relevance of the sample, docking simulations were also performed with temozolomide, a drug routinely used in the treatment of glioma. This additional analysis aimed to evaluate the binding potential of temozolomide to the *GATA2* wild-type and p.Arg396Trp-mutated proteins, providing a clinically relevant reference point for comparison with the other compounds investigated.

CB-Dock2 independently detected binding cavities on the AlphaFold-modeled p.Arg396Trp-mutated *GATA2* protein, including those in proximity to the mutation site. The results, along with the identified cavities and their corresponding binding conformations, are presented in Table 3.

A visual representation of the interactions between the compounds and the residues of *GATA2*, for both the wild-type and p.Arg396Trp-mutated proteins, is provided in Table 4. The table serves to elucidate differences in binding patterns and affinities between the wild-type and mutated protein models, offering insights into how the p.Arg396Trp mutation impacts drug binding. This analysis offers essential insights into the effects of structural alterations caused by the mutation on drug binding and underscores possible strategies for targeted therapeutic enhancement.

The docking scores revealed significant variations in binding affinities between the wild-type and mutated proteins. Entrectinib exhibited the strongest binding affinity for both wild-type (−10.5 kcal/mol) and mutant (−9.8 kcal/mol) *GATA2*. The interactions at the binding site exhibited alterations in residues adjacent to the mutation, specifically at ARG396 in the wild-type, which transitioned to TRP396 in the mutant. The docking score for Rucaparib marginally declined from −8.9 kcal/mol (wild-type) to −8.3 kcal/mol (mutant), exhibiting comparable residue interactions but nuanced alterations in hydrogen bonding near TRP396.

Vemurafenib exhibited similar binding affinities for the wild-type (−9.4 kcal/mol) and mutant (−9.0 kcal/mol) proteins, with alterations noted at the mutation site. Brigatinib exhibited negligible differences in binding scores between the wild-type (−9.1 kcal/mol) and mutant (−9.3 kcal/mol), preserving stable interactions despite the presence of TRP396 in the mutant configuration. For the clinically significant drug Temozolomide, the binding affinity was inferior relative to the other drugs, with values of −5.9 kcal/mol for wild-type and −6.1 kcal/mol for mutant *GATA2*. The interaction profile indicated slight alterations in residues adjacent to the mutation. These results indicate that the p.Arg396Trp mutation modifies the binding dynamics of *GATA2*, affecting drug affinity and implying potential consequences for drug efficacy and therapeutic approaches in glioma treatment.

Molecular docking analysis revealed computational changes in binding affinity between wild-type and mutant *GATA2* protein for several anti-neoplastic agents. The observed changes in AutoDock Vina scores represent computational predictions that require experimental validation through biochemical binding assays and functional drug sensitivity testing. Notable predicted changes included altered binding dynamics with Entrectinib, Rucaparib, Vemurafenib, and Brigatinib, though the clinical significance of these computational predictions remains to be determined. No statistical analysis was performed on docking scores as this represents a single-case computational study

## 4. Discussion

Whole-genome sequencing facilitates the detection of mutations, structural variants, and alterations in copy number throughout both coding and non-coding regions, thereby augmenting our comprehension of glioma biology. Upon further observation, the genes that show loss in our analysis are not those typically associated with poor prognosis. This aligns with the fact that our sample is an *IDH*-mutant glioma grade 2, which is generally associated with a better prognosis. According to the literature, wild-type gliomas, especially those categorized as *IDH* wild-type, frequently correlate with poorer prognoses relative to their *IDH*-mutant counterparts. This correlation is highlighted by various genetic modifications commonly found in these tumors, including losses of essential tumor suppressor genes such as *CDKN2A* and *CDKN2B*, alongside amplifications of oncogenes like *EGFR* [44,45]. Consistent with this, our sample does not show any losses in these genes

The *GATA2* gene encodes a transcription factor essential for multiple biological processes, especially hematopoiesis, the development of blood cellular components. *GATA2* is crucial for the preservation and differentiation of hematopoietic stem cells (HSCs) and progenitor cells, impacting their destiny and functionality [46,47]. It regulates genes essential for the development of various blood cell lineages, including myeloid and lymphoid cells [47,48]. The expression of the gene is meticulously regulated, as both overexpression and underexpression can result in considerable pathological effects.

Mutations in the *GATA2* gene can result in a range of diseases, predominantly marked by immunodeficiency and hematological disorders. *GATA2* deficiency is linked to multiple clinical manifestations, such as increased susceptibility to infections, hematological malignancies, and bone marrow failure syndrome [49,50,51]. Individuals with *GATA2* mutations may suffer from severe opportunistic infections due to compromised immune responses, particularly impacting natural killer (NK) cells and other elements of the innate immune system [50,52,53]. The deficiency may also result in conditions like monocytopenia and mycobacterial infections, collectively referred to as MonoMAC syndrome [53].

The association between *GATA2* and cancer is especially significant. *GATA2* mutations are associated with a heightened risk of myelodysplastic syndromes (MDS) and acute myeloid leukemia (AML) [48,51,54]. *GATA2* mutations contribute to leukemogenesis through mechanisms such as haploinsufficiency, wherein diminished *GATA2* levels inadequately regulate hematopoietic differentiation, resulting in the proliferation of aberrant cells [51,55]. Particular mutations, including gT354M and gR396Q, have been demonstrated to interfere with standard gene expression patterns and chromatin occupancy, thereby facilitating malignant transformation [48,51].

Furthermore, *GATA2’s* function transcends hematopoiesis; it is also involved in the regulation of additional developmental processes. *GATA2* participates in the differentiation of endothelial cells and has been demonstrated to inhibit cardiac differentiation in mesodermal progenitors [56,57]. This underscores the gene’s diverse function in both normal physiology and pathological conditions.

Recent studies have established *GATA2* involvement in brain tumor biology through multiple mechanisms. Fu et al. demonstrated that *GATA2* regulates constitutive *PD-L1* and *PD-L2* expression in brain tumors, suggesting its role in immune evasion [14]. Wang et al. showed that *GATA2* promotes glioma progression through *EGFR/ERK/Elk-1* pathway activation, establishing its oncogenic potential. These findings provide a mechanistic framework for understanding how *GATA2* mutations might contribute to glioma pathogenesis through altered transcriptional regulation of key oncogenic and immune regulatory pathways [15]. Our computational predictions suggest that the p.Arg396Trp mutation may alter these regulatory functions, though experimental validation is required to confirm these hypotheses.

Furthermore, the correlation of *GATA2* with additional oncogenic pathways in gliomas has been investigated. The gene MECOM, associated with poor prognosis in glioblastoma, has been demonstrated to interact with *GATA2* and other transcription factors [58]. This interaction indicates that *GATA2* may be integral to a wider network of regulatory elements that affect glioma biology and patient outcomes. Moreover, *GATA2* has been recognized as a crucial factor in the regulation of genes associated with brain stem cell biology, thereby underscoring its involvement in glioma pathogenesis [59].

While the specific p.Arg396Trp variant has not been previously reported, pathogenic *GATA2* mutations are well-characterized in hematologic malignancies, where they typically disrupt zinc finger domain function and DNA binding capacity. The arginine at position 396 is highly conserved across species and located within a critical functional domain, supporting the predicted pathogenicity of this substitution. The p.Arg396Trp mutation in the *GATA2* gene is a significant variant linked to multiple hematological disorders, especially those characterized by haploinsufficiency. *GATA2*, a transcription factor essential for hematopoiesis and immune function, has been associated with a range of clinical manifestations, including immunodeficiency, myelodysplastic syndromes (MDS), and acute myeloid leukemia (AML) [60,61]. The mutation specifically impairs the protein’s capacity to bind DNA, resulting in diminished transcriptional activity and subsequent haploinsufficiency, a prevalent mechanism in the pathophysiology of *GATA2*-related disorders [62,63].

Studies demonstrate that mutations such as p.Arg396Trp can result in a substantial reduction in *GATA2* expression, approximately 42% lower in affected individuals relative to controls [64]. This decrease corresponds with observations in animal models, where *GATA2* haploinsufficiency led to reduced *GATA2* levels in hematopoietic stem cells, thereby reinforcing the role of *GATA2* in sustaining normal hematopoiesis [64]. The occurrence of recurrent mutations at CpG dinucleotides, such as p.Arg396Trp, indicates a mutational hotspot potentially resulting from spontaneous deamination processes, underscoring the genetic instability linked to *GATA2* mutations [60].

Clinically, *GATA2* mutations are associated with various hematological malignancies. Patients with *GATA2* haploinsufficiency frequently exhibit MDS or AML, with a considerable percentage manifesting these disorders by the age of 40 [55]. Studies indicate that *GATA2* deficiency significantly influences leukemogenesis by establishing a conducive environment for further mutations, thus promoting the advancement to malignancy [63]. The correlation between *GATA2* mutations and opportunistic infections, along with other systemic complications, highlights the significant impact of *GATA2*-related disorders on patient health [50,53].

Gliomas frequently exhibit mutations in IDH within their genetic landscape. *IDH* mutations predominantly impact metabolic pathways but may also engage with transcription factors such as *GATA2*, thereby affecting tumor behavior and therapeutic responses [65]. The relationship between metabolic changes and transcriptional regulation in gliomas is intricate and requires additional research. In a clinical setting, significant findings reveal that TERT promoter (*TERTp*) mutations are prevalent in approximately 80% of glioblastomas, facilitating telomere maintenance and cancer advancement [66]. Mutations in *IDH1* and *IDH2* are essential for glioma classification and prognosis [67]. The Cancer Genome Atlas (TCGA) has delineated molecular subtypes of gliomas according to genetic modifications, which correlate with clinical outcomes [68]. Recent advancements in sequencing technologies, particularly nanopore sequencing, facilitate the simultaneous identification of essential prognostic markers, including *MGMT* methylation and *IDH* mutations, from clinical samples, thereby improving personalized treatment strategies [69].

Our findings in drug affinity analysis suggested the p.Arg396Trp mutation in *GATA2* alters its binding dynamics, leading to changes in the protein’s interaction with ligands and potentially modifying its role in regulatory pathways. This shift in binding properties may influence drug affinity, suggesting a direct impact on the efficacy of therapeutic agents targeting *GATA2*-associated pathways. Such findings highlight the need to consider this mutation in the context of glioma treatment strategies, as it may affect the response to existing therapies and pave the way for designing mutation-specific interventions to improve therapeutic outcomes.

To validate and refine the findings from DrugRep, CB-Dock2, a robust docking tool developed by the Yang Cao Lab, was employed. CB-Dock2 autonomously identified binding cavities on the AlphaFold-modeled *GATA2* protein structure, including regions adjacent to the p.Arg396Trp mutation site. Using this platform, flexible docking simulations were conducted for the highest-ranking compounds identified by DrugRep. Additional docking simulations focused on compounds that exhibited differential binding affinities between the wild-type and mutant *GATA2* proteins, providing further insights into the functional impact of the mutation on drug interaction dynamics [22,23].

From these analyses, compounds demonstrating the highest binding affinities and stable interactions were prioritized for further consideration. Special attention was given to FDA-approved drugs and those currently undergoing clinical trials to streamline their potential for repurposing. By integrating DrugRep’s receptor-based screening capabilities with CB-Dock2’s cavity-specific docking validation, a synergistic workflow was achieved. DrugRep efficiently screened a vast library of compounds to identify high-affinity candidates, while CB-Dock2 confirmed these results and provided deeper insights into the specific molecular interactions at the mutation site.

This dual approach successfully identified promising therapeutic candidates targeting the mutated *GATA2* protein in glioma. The workflow not only emphasized precision in virtual screening but also highlighted the potential for accelerated drug repurposing. Furthermore, the combination of advanced screening and structural modeling techniques underscores a streamlined and reproducible strategy for prioritizing drug candidates in the context of glioblastoma and other mutation-driven diseases. These findings establish a foundation for future experimental validation and potential clinical translation, reinforcing the utility of in silico methods in precision oncology.

### 4.1. Limitations

This study has several important limitations that must be acknowledged. Our analysis is based on a single clinical case, which significantly limits the statistical power and generalizability of our findings. The biological relevance of the *GATA2* p.Arg396Trp mutation in glioma pathogenesis cannot be established without validation in larger patient cohorts. The drug affinity changes represent computational predictions that require confirmation through biochemical binding assays and functional drug sensitivity testing. The clinical significance of this mutation for patient prognosis and therapeutic response remains unknown and requires long-term follow-up studies in additional cases. We did not perform independent confirmation of the mutation using Sanger sequencing or assess its impact on *GATA2* protein expression and function in glioma cells.

### 4.2. Future Directions

Future studies should focus on experimental validation of our computational predictions through multiple approaches. Sanger sequencing confirmation of the mutation in additional tissue samples would strengthen the genetic findings. Functional assays, including luciferase reporter assays, chromatin immunoprecipitation, and RNA sequencing, could elucidate the transcriptional consequences of the mutation. Cell viability assays and drug sensitivity testing using patient-derived glioma cells would validate the predicted therapeutic implications. Additionally, screening larger cohorts of lower-grade glioma patients for *GATA2* mutations could establish the frequency and clinical significance of such alterations in this tumor type.

## 5. Conclusions

We report the first identification of a *GATA2* p.Arg396Trp mutation in a lower-grade glioma patient. Our comprehensive computational analysis suggests this variant may be pathogenic and could influence transcriptional regulation and drug binding affinity. However, these findings represent preliminary observations from a single case that require extensive experimental validation and clinical correlation in larger patient cohorts. While our study provides a foundation for future investigations into the *GATA2* role in glioma biology, the clinical significance of this mutation remains to be established through functional studies and expanded clinical series.

## Figures and Tables

**Table 1:** Pathogenic variants identified in genetic analysis.

Gene(s)	Significance	Type	Consequence	HGVSc	HGVSp	Previous Association	Reference
*PERM1*	Pathogenic	SNV	Missense variant	NM_001291366.2:c.2330T>C	p.Val777Ala	Mitochondrial biogenesis	Cho et al. [25]
*MTHFR*	Pathogenic	SNV	Nonsense	NM_001330358.2:c.1531G>T	p.Glu511 *	Folate metabolism, glioma risk	Bethke et al. [26]
*APOA2*	Pathogenic	SNV	No consequences found	NM_001643.2:c.-323C>T	-	Lipid metabolism	Knott et al. [27]
*HKDC1*, *LOC101928994*	Pathogenic	SNV	Missense variant	NM_025130.4:c.173C>T	p.Thr58Met	Glucose metabolism	Irwin & Tan [28]
*ADRA2A*	Pathogenic	SNV	3′ UTR variant	NM_000681.4:c. *427A>G	-	Adrenergic receptor	Kobilka et al. [29]
*SMPD1*	Pathogenic	SNV	No consequences found	NM_000543.5:c.911T>C	p.Leu304Pro	Lysosomal enzyme	Schuchman et al. [30]
*GNG3*, *BSCL2*, *HNRNPUL2-BSCL2*	Pathogenic	SNV	Intron variant	NM_012202.5:c.-431C>T	-	G-protein signaling	Agarwal et al. [31]
*SYCP3*	Pathogenic	Deletion	Frameshift variant	NM_001177948.2:c.643delA	p.Ile215fs	DNA repair, meiosis	Yuan et al. [32]
*HNF1A*	Pathogenic	Deletion	Frameshift variant	NM_000545.8:c.872delC	p.Pro291fs	Transcription factor	Kavitha et al. [33]
*GJB2*	Pathogenic	SNV	Missense variant	NM_004004.6:c.109G>A	p.Val37Ile	Gap junction protein	Kelsell et al. [34]
*MAPK7*	Pathogenic	SNV	Missense variant	NM_139032.3:c.469G>A	p.Ala157Thr	MAPK signaling	Lee et al. [35]
*AVPR2*	Pathogenic	Deletion	Frameshift variant, Non-coding transcript variant	NM_000054.7:c.738delG	p.Arg247fs	Hormone receptor	Rosenthal et al. [36]
*IDH1*	Pathogenic/likely pathogenic	SNV	Missense variant	NM_001282386.1:c.395G>A	p.Arg132His	Well-established driver	Yan et al. [37]
*GATA2*	Pathogenic/likely pathogenic	SNV	Missense variant	NM_001145661.2:c.1186C>T	p.Arg396Trp	Not previously reported	This study
*TP53*	Pathogenic/likely pathogenic	SNV	Splice donor variant	NM_000546.6:c.559+1G>T	-	Tumor suppressor	TCGA [38]

* stop codon.

**Table 2:** Domain identification analysis.

Interpro Domain	Gene Ontology Term	Broad Gene Ontology Term
Zinc Finger, Gata-Type IPR000679	Sequence-Specific DNA-Binding GO:0043565	Binding GO:0005488 Molecular_Function GO:0003674 Nucleic Acid Binding GO:0003676
Zinc Finger, Nhr/Gata-Type IPR013088	Zinc Ion-Binding GO:0008270	Molecular_Function GO:0003674 Binding GO:0005488 Ion Binding GO:0043167
Transcription Factor, Gata-2/3 IPR016374	DNA-Binding Transcription Factor Activity, Rna Polymerase Ii-Specific GO:0000981	Molecular_Function GO:0003674
Transcription Factor Gata IPR039355	DNA-Binding Transcription Factor Activity GO:0003700	Molecular_Function GO:0003674

**Table 3:** Vina score of the molecular docking parameter for selected anti-neoplastic compounds.

Drug/Compound	Wild-Type *GATA2*		p.Arg396Trp-Mutated *GATA2*	
	Vina	Dock Chain	Vina	Dock Chain
Entrectinib C_31_H_34_F_2_N_6_O_2_	−10.5	Chain A: GLU294 CYS295 VAL296 ASN297 CYS298 ASP309 HIS313 LEU315 GLN328 ASN329 ARG330 PRO331 LEU332 ILE333 LYS334 PRO335 LYS336 ARG337 ARG338 SER340 ALA342 THR358 ILE393 GLN394 THR395 **ARG396** ASN397 ARG398 LYS399 MET400 SER401 ASN402	−9.8	Chain A: VAL296 ASN297 CYS298 ASP309 THR311 HIS313 LEU315 GLN328 ASN329 ARG330 PRO331 LEU332 ILE333 LYS334 PRO335 LYS336 ARG337 ARG338 LEU339 SER340 ALA342 THR358 ASN371 ILE393 GLN394 THR395 **TRP396** ASN397 ARG398 LYS399 MET400 SER401 ASN402
Rucaparib C_19_H_18_FN_3_O	−8.9	Chain A: GLU294 CYS295 VAL296 ASN297 CYS298 ASP309 THR311 HIS313 LEU315 HIS323 GLN328 ASN329 ARG330 PRO331 LEU332 ILE333 LYS334 PRO335 LYS336 ARG338 SER340 THR358 THR395 **ARG396** ASN397 ARG398 LYS399 MET400 SER401 ASN402	−8.3	Chain A: VAL296 ASN297 CYS298 ASP309 THR311 HIS313 LEU315 HIS323 GLN328 ASN329 ARG330 PRO331 LEU332 LYS334 PRO335 LYS336 ARG337 ARG338 LEU339 SER340 THR358 ILE393 GLN394 THR395 **TRP396** ASN397 ARG398 LYS399 MET400 SER401
Vemurafenib C_23_H_18_ClF_2_N_3_O_3_S	−9.4	Chain A: GLU294 VAL296 ASN297 CYS298 GLY299 ALA300 THR301 ALA302 ASN329 ARG330 LEU332 LYS334 PRO335 LYS336 ARG337 ARG338 LEU339 SER340 ALA342 ARG343 THR356 THR357 THR358 ILE393 GLN394 THR395 **ARG396** ASN397 ARG398 LYS399 MET400	−9.0	Chain A: GLU294 VAL296 ASN297 CYS298 ASP309 HIS313 LEU315 GLN328 ASN329 ARG330 PRO331 LEU332 LYS334 PRO335 LYS336 ARG337 ARG338 SER340 THR358 GLN394 THR395 **TRP396** ASN397 ARG398 LYS399 MET400 SER401 ASN402
Brigatinib C_29_H_39_C_l_N_7_O_2_P	−9.1	Chain A: VAL296 ASN297 CYS298 ASP309 THR311 HIS313 GLN328 ASN329 ARG330 PRO331 LEU332 LYS334 PRO335 LYS336 ARG337 ARG338 LEU339 SER340 ALA342 THR358 THR395 **ARG396** ASN397 ARG398 LYS399 MET400 SER401 SER404	−9.1	Chain A: GLY292 GLU294 VAL296 ASN297 CYS298 ASP309 THR311 GLY312 HIS313 TYR314 GLN328 ASN329 ARG330 PRO331 LEU332 LYS334 PRO335 LYS336 ARG337 ARG338 LEU339 SER340 ALA342 THR357 THR358 ILE393 THR395 **TRP396** ASN397 ARG398 LYS399 MET400 SER401 SER404
Temozolamide C_6_H_6_N_6_O_2_	−5.9	Chain A: CYS352 THR354 THR356 THR357 THR358 LEU359 CYS370 ASN371 ALA372 CYS373 ARG384 MET388 LYS389 LYS390 GLU391 GLY392 ILE393 GLN394	−6.1	Chain A: CYS352 THR354 THR356 THR357 THR358 LEU359 CYS370 ASN371 ALA372 ARG384 MET388 LYS389 LYS390 GLU391 GLY392 ILE393 GLN394 **TRP396**

**Table 4:** The visual representation of the interactions between the compounds and the residues of *GATA2*.

Drug/Compound	Wild-Type *GATA2*	p.Arg396Trp-Mutated *GATA2*
Entrectinib C_31_H_34_F_2_N_6_O_2_	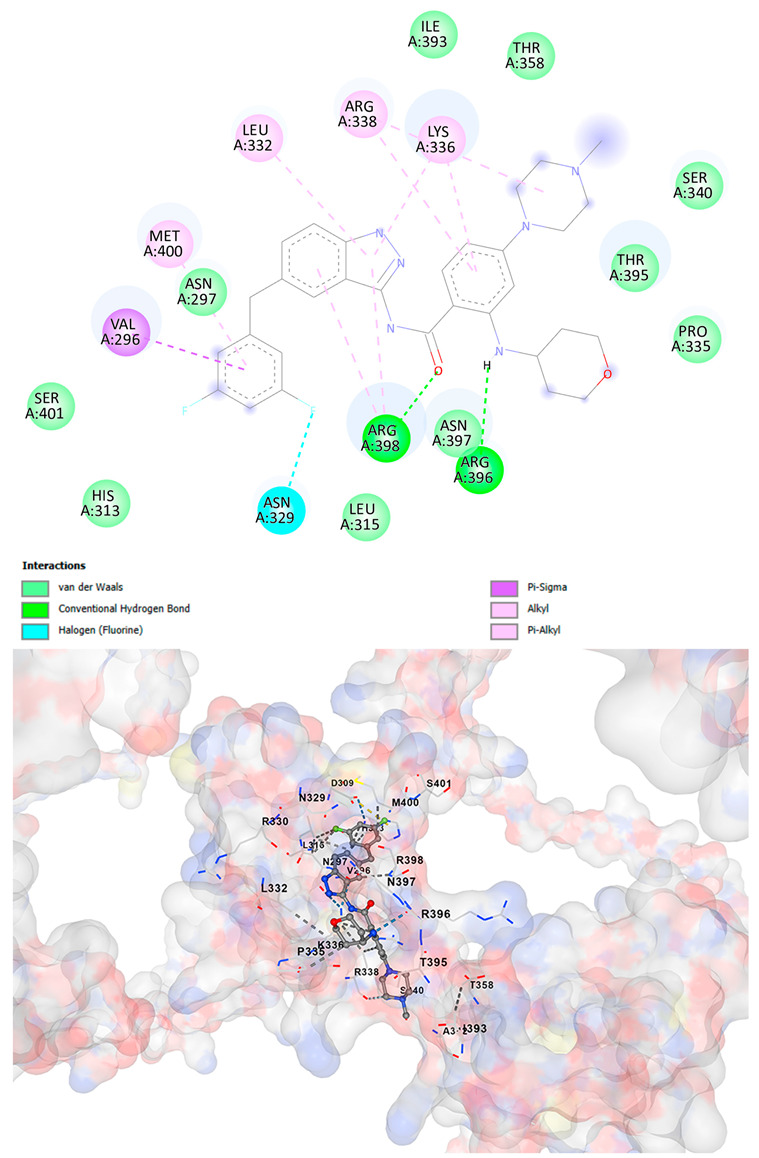	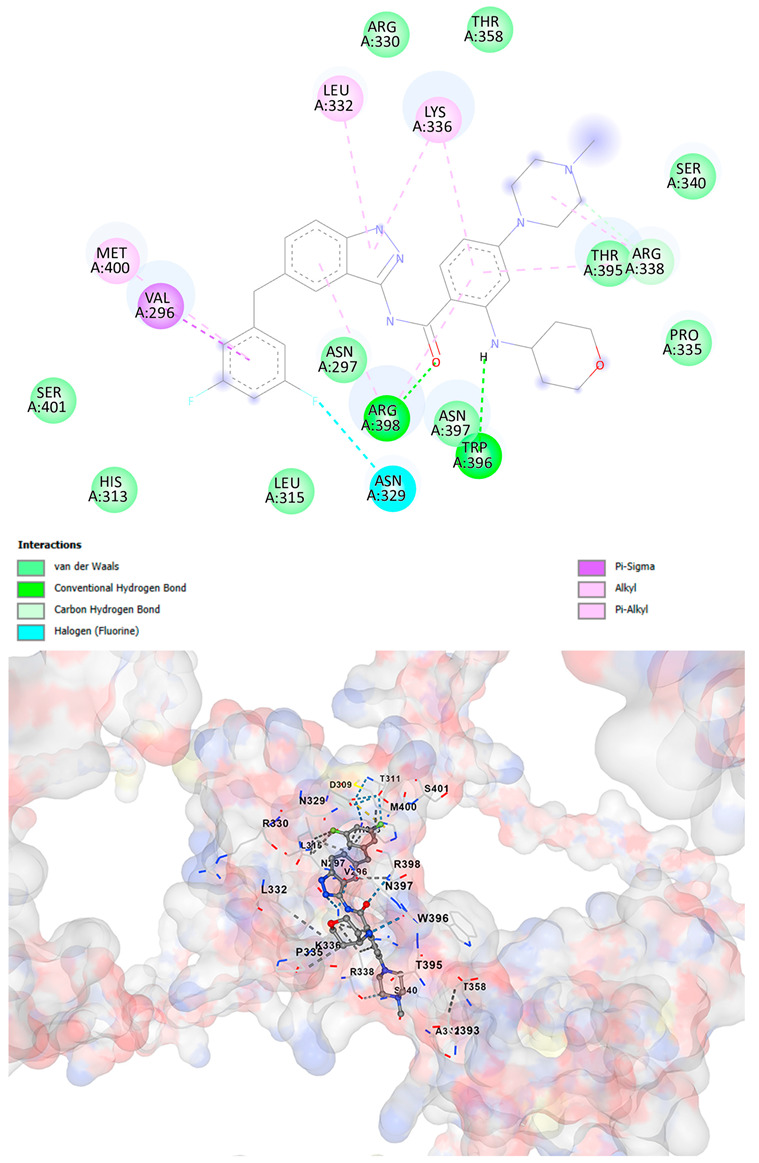
Rucaparib C_19_H_18_FN_3_O	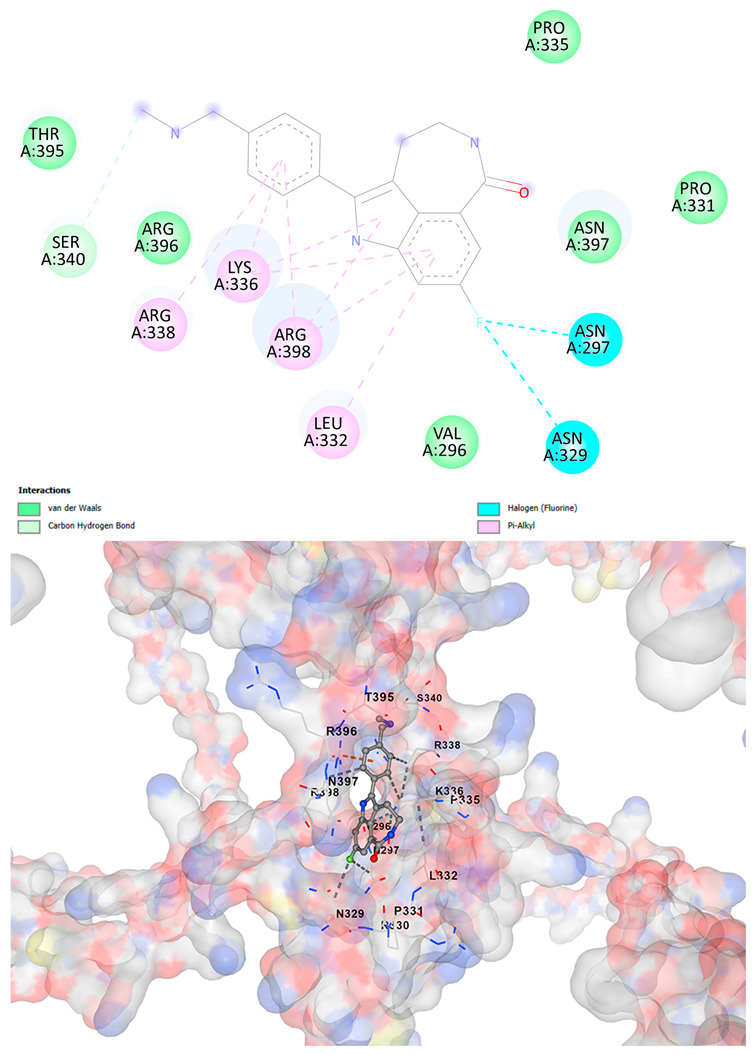	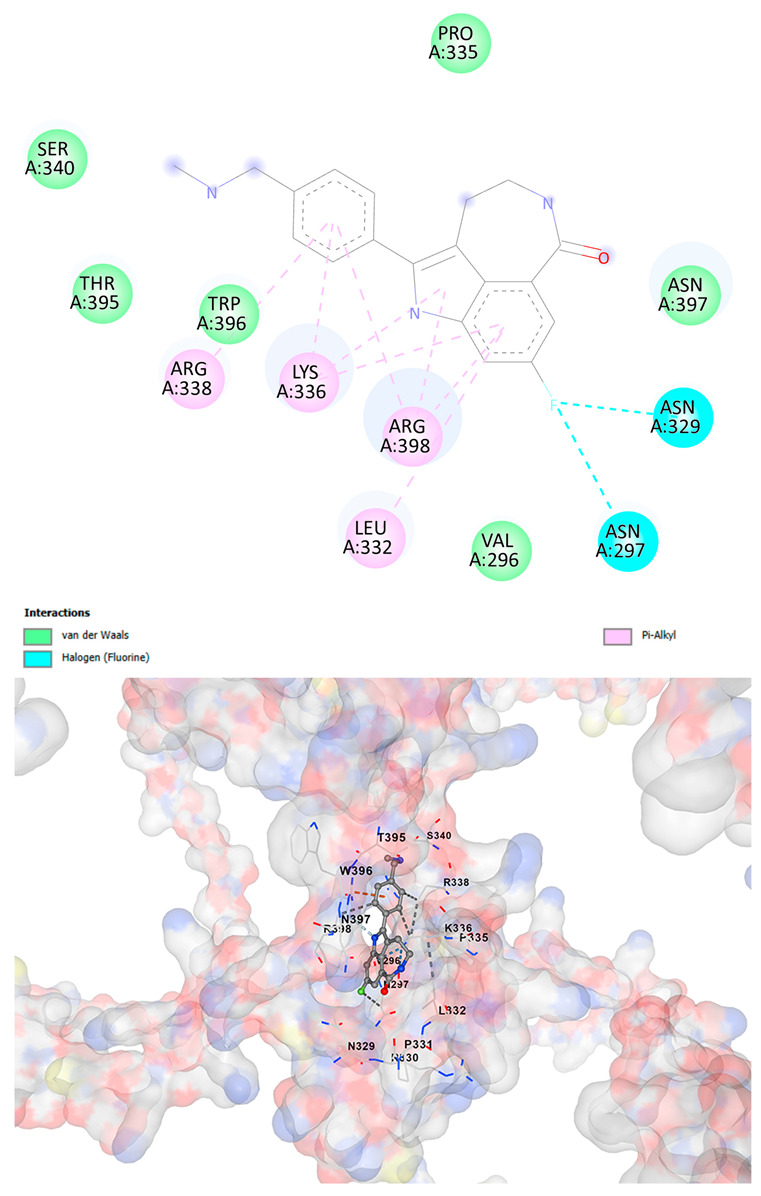
Vemurafenib C_23_H_18_ClF_2_N_3_O_3_S	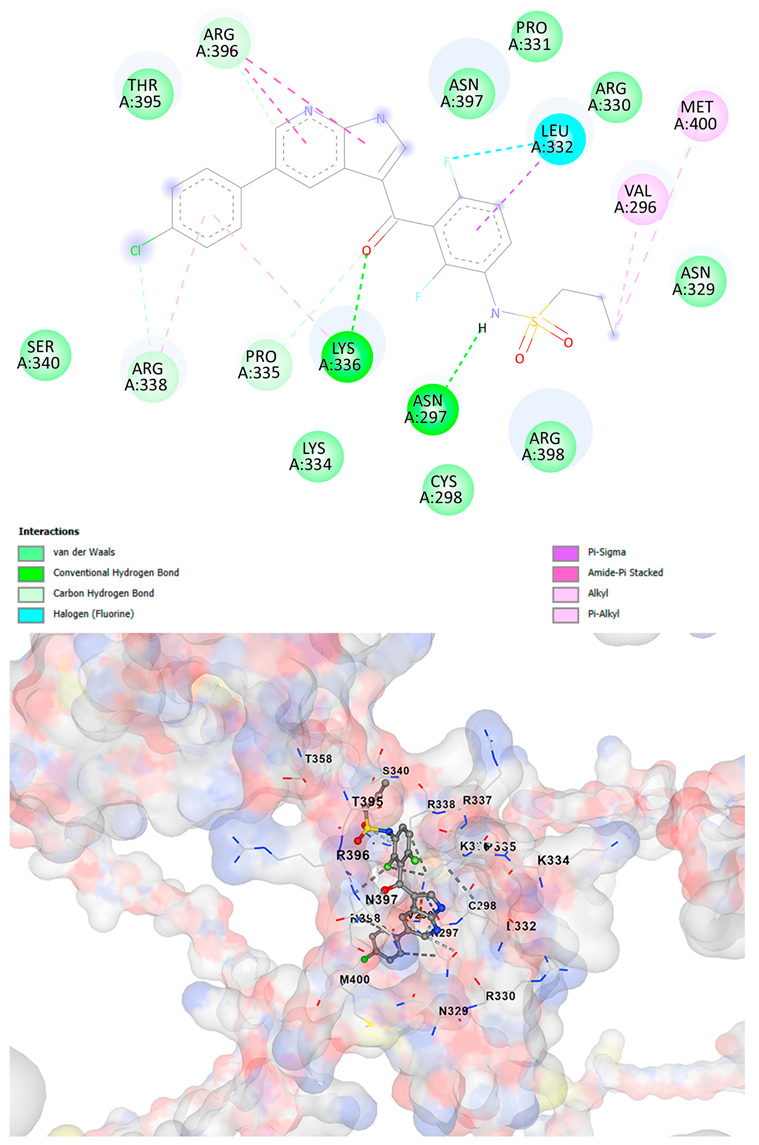	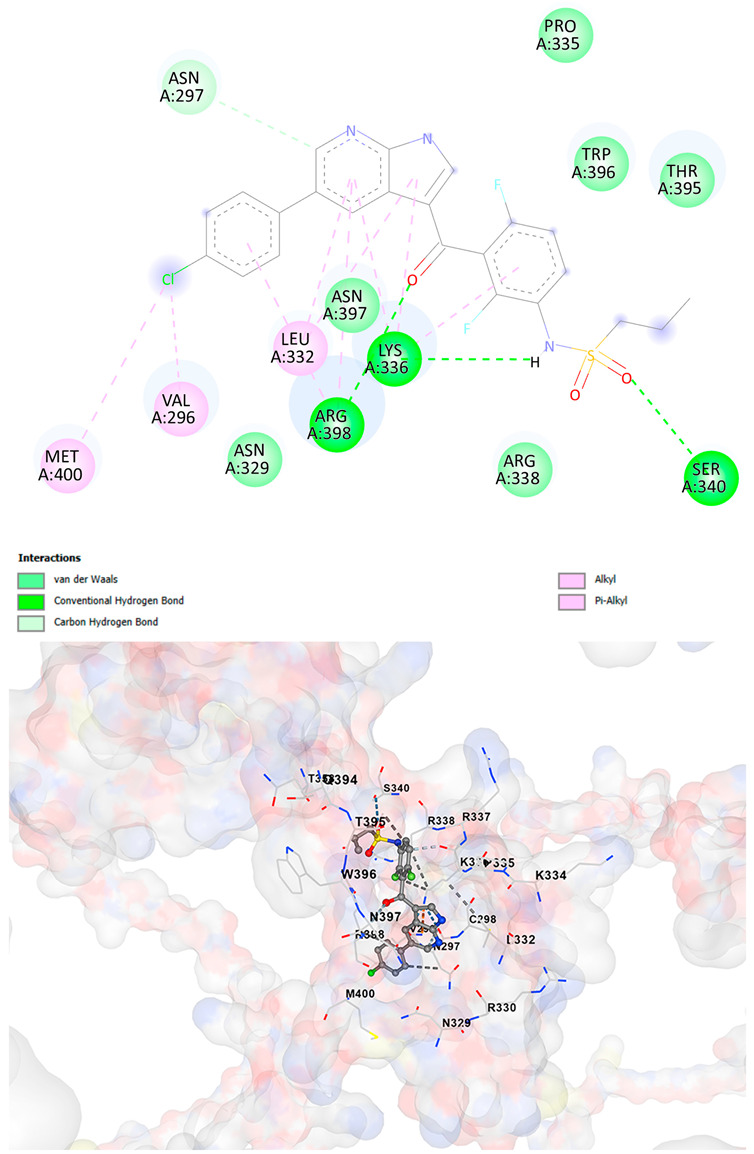
Brigatinib C_29_H_39_C_l_N_7_O_2_P	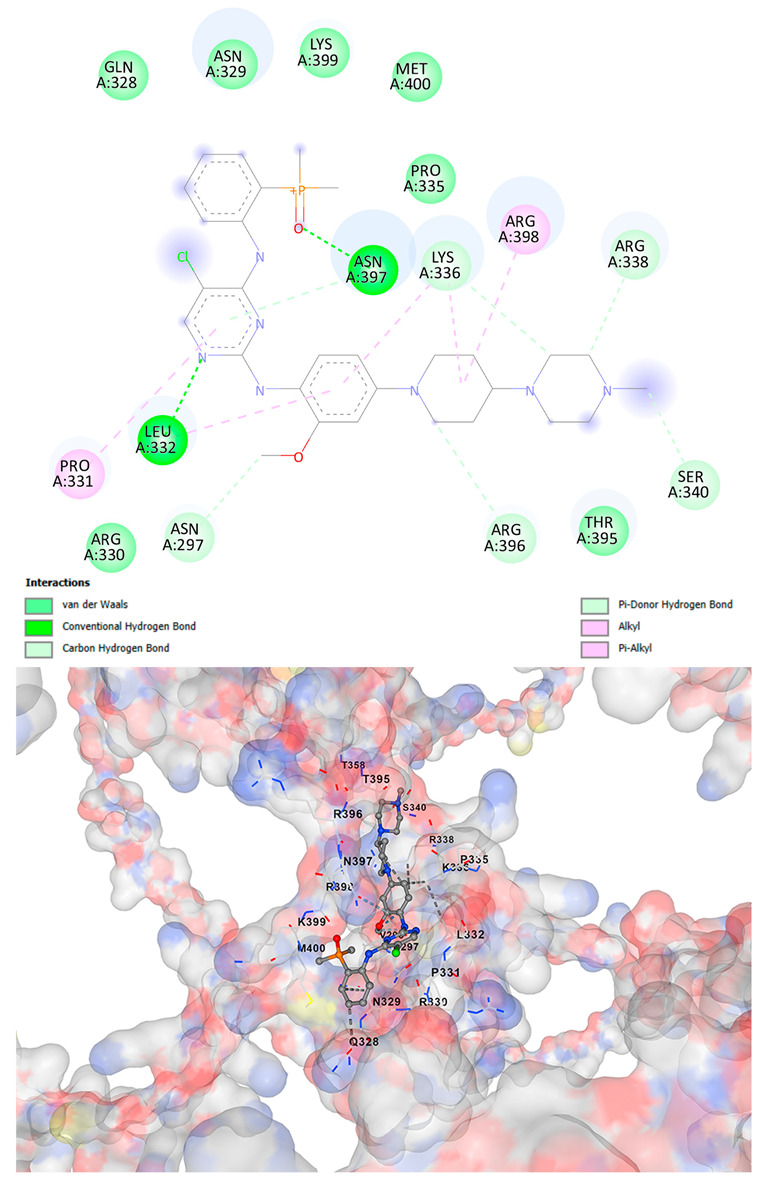	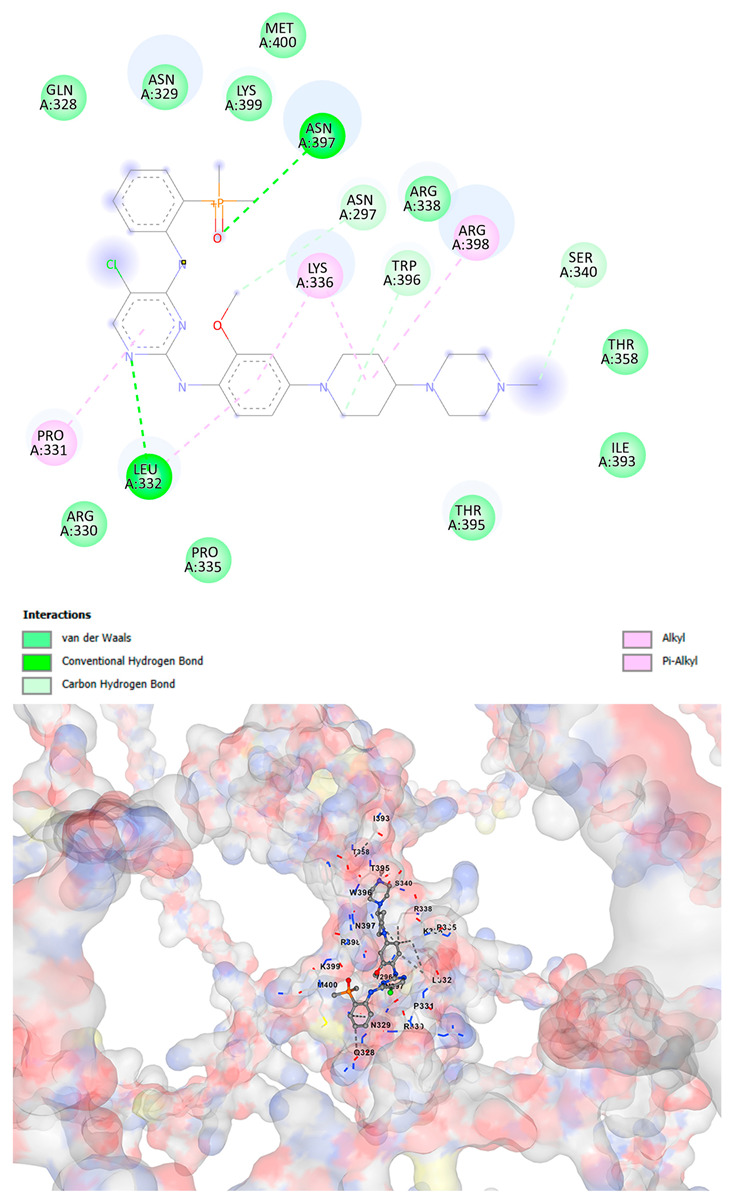
Temozolamide C_6_H_6_N_6_O_2_	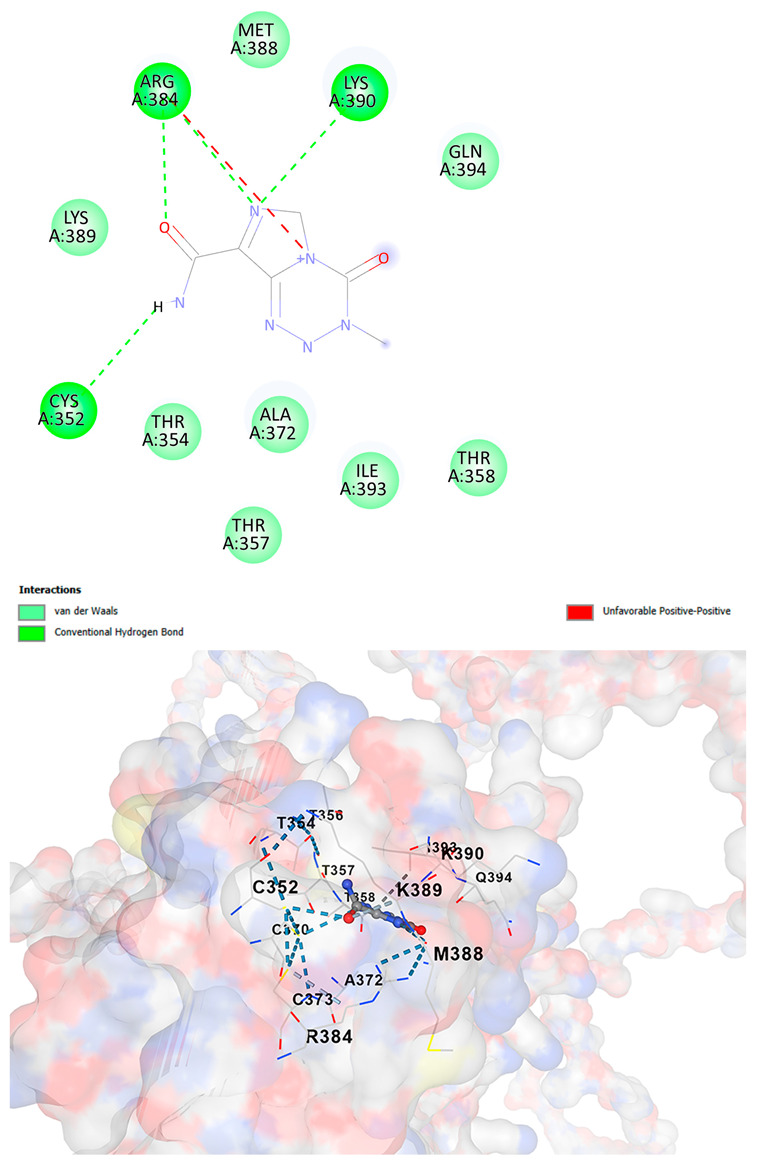	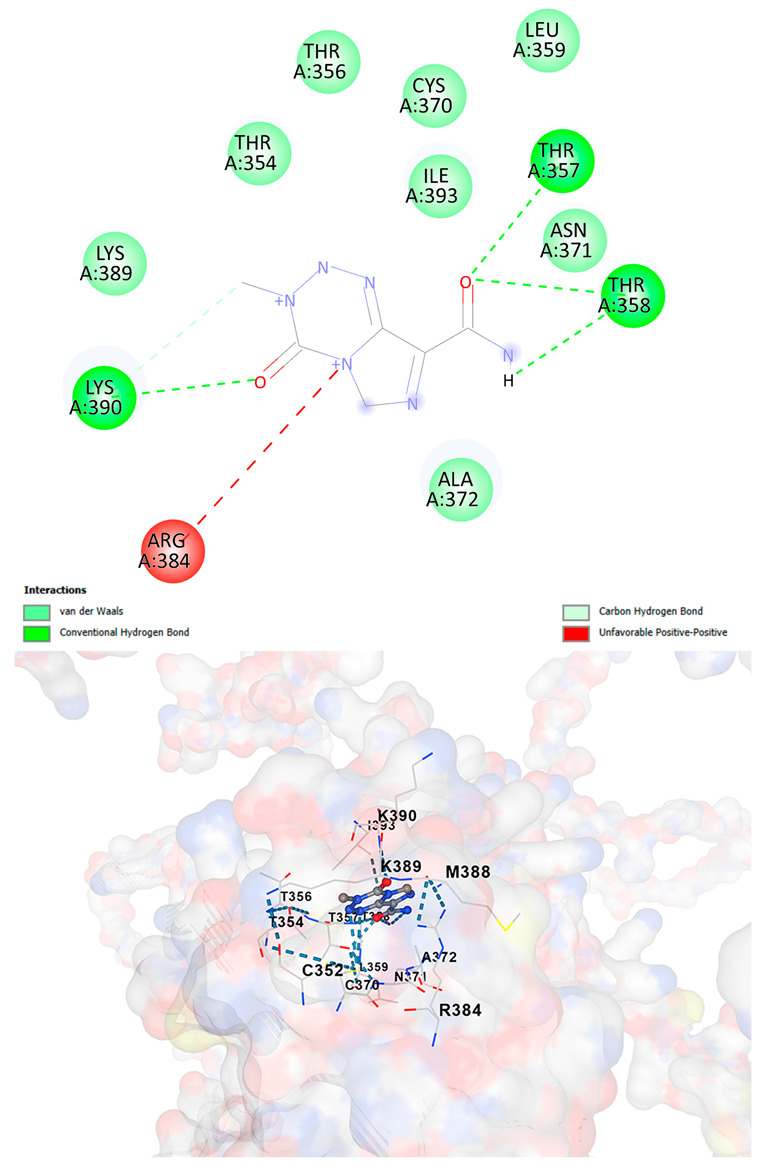

## Data Availability

The original contributions presented in this study are included in the article. Further inquiries can be directed to the corresponding authors.

## Supplementary Materials

The following supporting information can be downloaded at: https://www.mdpi.com/article/10.3390/cancers17203338/s1. Table S1. Single-nucleotide variants—detailed information.

## Abbreviations

The following abbreviations are used in this manuscript:

AML	Acute Myeloid
CNV	Copy Number Variation
IDH	Isocitrate Dehydrogenase
KEGG	Kyoto Encyclopedia of Genes and Genomes
MDS	Myelodysplastic Syndrome
PBS	Phosphate-Buffer Saline
SNV	Single Nucleotide Variants
WGS	Whole-Genome Sequencing
